# Forensic child and Adolescent Psychiatry and mental health in Europe

**DOI:** 10.1186/1753-2000-5-20

**Published:** 2011-06-29

**Authors:** Theo AH Doreleijers, Jörg M Fegert

**Affiliations:** 1 VU University Medical Center Amsterdam, PO Box 303, 1115 ZG Duivendrecht, The Netherlands; 2 Leiden University, Law School Leiden University, Leiden, The Netherlands; 3 University Hospital Ulm, Department of Child and Adolescent Psychiatry/Psychotherapy, Steinhövelst 5, 89075 Ulm, Germany

## Editorial

The medical and educational interests of young people who find themselves in the police and justice systems are the main aims of the European Association for Forensic Child and Adolescent Psychiatry, Psychology and other involved professions, EFCAP-EU, which was officially founded in 1997. Two years earlier the first youth forensic symposium was held at the ESCAP Congress in Utrecht, containing eight papers from all over Europe. Other aims of EFCAP are:

- to improve forensic assessment and treatment of children and adolescents in the justice system, as well as of their families

- to improve facilities for these young people

- to facilitate joint international research, and

- to promote international training and education.

EFCAP-EU is a federation of some officially founded national EFCAP's like in Finland, Switzerland and The Netherlands, and of working groups in other countries like Germany (Arbeitsgemeinschaft), United Kingdom (unit of the Royal College of Psychiatrists), Italy, Portugal, Spain, Hungary and Turkey. Belgium, Italy, Luxembourg and France are initializing a national EFCAP. Other countries sympathize with EFCAP without having yet formal organizations: Sweden, Norway, Austria, and Ukraine.

EFCAP's activities focus on:

- Since 1995 annual conferences organized in collaboration with EAPL, ESCAP, IALMH; since 2008 biannual EFCAP-congresses on its own: 2008 Amsterdam, 2010 Basel, 2012 Berlin;

- Educational exchange: Finnish and British colleagues came to Holland, Dutch colleagues went to the United Kingdom, Italy and Germany;

- Student projects: Dutch student-trainees in Italy, Spain and UK, a German student in The Netherlands

- Phd-projects: Dutch phd-student in Germany and UK; PhD-ceremonies in Belgium, Norway and Sweden; Finnish research in Belgium and Holland; Swiss PhD-project with Germany and The Netherlands;

- Practice experiences exchange: Russia, Ukraine.

Striking and topical themes at the EFCAP 2010 Basel Congress were focusing on specific groups like children and adolescents with characteristics of psychopathy, offenders of very young age, juvenile sex offenders and girls in the justice system. There were quite a few presentations on neurobiological research topics, on intercultural issues and on risk assessment and management in juveniles. What is more, practice presentations were given: individual interventions, family therapies, restoration and mediation. In addition there were also presentations on policy and legislation.

The Basel Congress was opened by an overview of what is going on in the countries represented in the board. A summary is given here below.

## What is going on in The Netherlands (Theo A. H. Doreleijers)?

Juvenile crime is decreasing. You never know what figures really mean, but annual similar registration methods elicited an ongoing decrease of youth crime. It explains also the sudden overcapacity of residential justice facilities: nowadays more than 50% of the juvenile justice capacity is not any more in use. Apart from crime decrease the tendency of magistrates to convict young people to a civil instead of a penal measure also caused this vacancy. On the other hand the residential youth care had to build new facilities to house all these new populations.

In the mean time the Ministry of Justice invests a lot of money and energy to improve the quality of interventions offered to young people under its roofs. A Committee for the Approval of Justice Interventions was set up some years ago. In the future, only officially approved interventions will be financed. And, finally: much more research is carried out in the Justice field: epidemiological and longitudinal studies, intervention effect studies, and even studies using modern techniques like fMRI. The national science foundation funded the initiative for an Academic Working Place for Forensic Youth Care with one million Euro's (collaboration of the Leiden and Amsterdam Universities Child and Adolescent's departments with two Juvenile Justice Institutions).

The new government has planned (to be elaborated in the upcoming years) a new Adolescent Criminal Law for youths between 15 and 23 years of age. Everyone is curious about this initiative: is it going to help young people or just make the measures and punishments more restrictive?

## What is going on in Spain? (Josep Cornella)

After some successful conferences in Gerona, Valencia, Seville and Tarragona (2010) with forensic topics a 'Controversial Session' will be held on the accountability of minors at the Spanish Pediatric Association Congress in Valladolid (June 2011) and a comparable session at the Social Pediatric Congress in Granada (October 2011).

## What is going on in Germany? (Renate Schepker and Jörg M. Fegert)

In the South of Germany a first course was organized for a certificate in Forensic Assessment. The State of Bavaria decided to found a forensic unit for adolescents in Regensburg. In legislation, the Law on Secure Detention has to be revised (due to a sentence of the European Court in Brussels). The Bundesarbeitsgemeinschaft der Leitenden Ärzte, Untergruppe Maßregelvollzug (representing the adolescent forensic units in Germany) of the German Child and Adolescent Psychiatry Society was asked to join the debate.

At the moment there is a lot of public and political debate about sexual abuse in institutions in Germany. For the first time research into these issues has been set up. Further on, there are many (usual) discussions about migrant youth offenders and how to react appropriately upon them in court and in the pedagogical system. Much attention deserved school shootings like in Winnenden and related copycats [1].

## What is going on in Portugal? (Antonio Castro Fonseca)

After the sexual abuse cases came to the public, there is a much stronger sensibility (in the public and in the courts, etc) regarding the children's neglect and sexual abuse [2,3]. A committee was founded for the protection of minors, attracted to each local authority and the youth re-education centres have been reorganized, which has led to the closing of several of them.

There is a new law which says that the same crime of sexual abuse committed by the same individual against the same victim is considered as a single continued crime. And a new student/pupil status (Estatuto do Aluno) was set up that give more power and facilities to the head of the schools to act and punish pupils bad behaviour.

## What is going on in Italy? (Marco Zanoli & Giovanni Camerini)

In Italy there is still not a spread culture of clinical evaluation of risk and protective factors in youngsters and there's no general consensus about prevention-rehabilitation interventions. Evaluations are done by social services and minor justice centers using criteria that are more 'social' than clinical.

## What is going on in Finland? (Riittakerttu Kaltiala-Heino)

The two massacres by shooting in schools in 2007 and 2008 have resulted in great concerns about access to psychiatric care of young people. The Ministry of Welfare and Health has supported the development of flexible services for child and adolescent mental health and welfare services by financing the development of projects aiming to enhance early recognition, prevention and early treatment. Research is initiated to identify psychopathology in kids who have been brought to psychiatric services due to threats of school massacres

## What is going on in Russia? (Елена Дозорцева)

Within the last 3 years the number of juveniles in 62 penitentiary institutions was reduced from 10,7 (2007) to 6 (2009) thousand. The total number of crimes perpetrated by juveniles or with participation of juveniles decreased from 139 (2007) to 95 (2009) thousands.

There is no national separate system of juvenile justice, but in the Rostov region function four juvenile courts. In many other regions specialization of judges in the framework of general criminal courts is implemented. In some courts so called 'juvenile technologies' like restorative justice procedures are realized. The situation with social perception of juvenile justice has worsened in the course of the last two years due to an organized campaign of its opponents. However, in February 2011 a special resolution of the Supreme Court of Russian Federation declared individual approach to juvenile offenders, their re-socialization, protection of their rights and prevention of juvenile crimes as main principles of the courts dealing with juveniles. The resolution gives the detailed practical interpretation of the principles and instructions for judges and specialists. In 2009-2010 in Moscow 75 students graduated from the educational program 'Juvenile Forensic Psychology'. The total number of students at the moment is more than 150. In 2011 a new program 'Pedagogy and Psychology of Deviant Behaviour' will be launched.

## What is going on in the United Kingdom? (Sue Bailey)

The establishment of Youth Offender Services has over this time led to much improved multi-agency services for offenders with mental-health problems. In particular the intensive wraparound services for those leaving custody have this year led to reduction in the number of young people in custody with an associated fall in juvenile offending and closure of some juvenile prison beds In the specific area of mental health Child and Adolescent Mental Health Service teams have now been embedded into every juvenile justice unit with special units for these adolescents with most challenging behaviour There are now 6 nationally commissioned adolescent psychiatric secure inpatient units, two of them for learning disabled kids. Despite challenging times adolescent forensic mental health in the UK is thriving with a renewed drive to have the age of criminal responsibility raised.

## What is going on in Switzerland? (Klaus Schmeck)

The first adolescent forensic inpatient unit in Basel will be opened next year. In Bern, Solothurn, St. Gallen, Geneva and Lausanne are smaller units as well. In the Swiss Society of Forensic Psychiatry there is a section of adolescent forensic psychiatry; a formal curriculum for the training in this subspecialty that is formally approved by the Swiss Medical Association has been started In Basel research with delinquent adolescents has been expanded to 62 institutions in all parts of Switzerland.

## What is going on in Belgium? (Dirk van West)

There are more closed facilities in Flandres and Wallony and there is a policy direction for 250 more places in adult facilities. Antwerp took a Functional Family Therapy Initiative; more exchange came up between child psychiatry and youth care. Shortly a Working Group on Forensic Psychiatry within the Flemish Society of Child and Adolescent Psychiatry was started.

## What do we learn from all this information?

The main tendency to be observed is that in many countries mental health services are taking over the care for disordered juvenile delinquents which resulted in lower crime figures and in an overcapacity of places in the justice field. In the mean time America-like school shootings are taking over the floor in Europe which makes politicians cry for more serious punishment.

EFCAP has a main role in taking up this challenge, by performing adequate research, by international exchange of practice experiences and education, by showing politicians and policy makers what the problems really stand for, and by developing new interventions. EFCAP Berlin, March 7th to 9th 2012 (http://www.efcap2012.de) will help us building a new future for these kids.

The articles in this special issue can also be considered as the result of the EFCAP 2010 conference in Basel, Switzerland. The article of Steiner et al. [4] examines the validity or usefulness of the categorization of aggressive behaviour in two subtypes: planned, instrumental, predatory (PIP) vs. reactive, affective, defensive, impulsive (RADI) aggression. The article therefore contributes to a better understanding of young offenders and presents implications for targeted psychopharmacological and other trauma specific interventions. Van Domburgh and colleagues [5] aimed at identifying risk factors for level of offending among children from different socio-economic status (SES) neighbourhoods and ethnic origins. The findings revealed that only few neighbourhood differences had an impact on individual and parental risk factors but individual and parental risk factors can differ between ethnic groups. The study of Veen and colleagues is the first one which examined [6] ethnic differences in the mother-son relationship of Moroccan and native Dutch incarcerated and non-incarcerated delinquent male adolescents in the Netherlands. The findings indicate that mother-son relationship types of incarcerated Moroccan adolescents and non-incarcerated Moroccan adolescents are rather comparable. The article of Doreleijers and colleagues [7] investigates the psychometric properties and the perceived usefulness of the Dutch: BAsis RaadsOnderzoek (BARO). The BARO is a first-line screening instrument and helps to identify psychiatric disorders, adverse environmental factors, and levels of (dys)function in adolescent offenders.

## 3rd EFCAP Congress in Berlin

This issue is to introduce some of the topics of next year's meeting of the European Association for Forensic Child and Adolescent Psychiatry, Psychology and other involved professions. As laid out, both authors are deeply involved with the aims and organization of this congress: Theo A. H. Doreleijers as the president of EFCAP and Jörg M. Fegert as next year's congress president. This congress will be held in Berlin, Germany, from 7 - 9 March 2012. As in Germany the legal framework defines a group of young adults that is dealt with in shared responsibility of adult psychiatrists as well as child and adolescents psychiatrists, we decided to focus on both groups: adolescents and young adults.

The main theme of the congress "Young Offenders and Victims - Forensic Psychiatry and Psychology in Children, Adolescents and Young Adults" reflects a broad discussion, held world wide, on traumatic influences and transgenerational cycles of violence. At the same time we want to focus more on studies and expertises on young victims. In Germany during the last year a public debate on child sexual abuse in educational institutions and in many other fields has started and sexual violence among adolescents is one of the highly debated topics.

The congress will take place at the newly restored Langenbeck-Virchow House, which was re-opened on 1 October 2005 by the German Surgery Association and the Berlin Medical Association. The building, which was first inaugurated in 1915, next to the famous Charité campus in the centre of Berlin, is now once again available to professional associations as a centre for medical and interdisciplinary exchange.

We hope to create interest in the readers of *CAPMH *to join other scientists and practitioners in 2012 in Berlin, to have interesting discussions and to learn from each other. Recent download statistics of *CAPMH *show that many manuscripts in this scientific journal come from all over the world. The majority of our readership comes from the United States of America, the UK, Germany, Canada, Australian, Sweden, Norway, the Netherlands, India and China. The European Association for Forensic Child and Adolescent Psychiatry, Psychology and other involved professions explicitly invites all those readers from non European countries to join us in our discussions in Berlin. At the Basel meeting we had important contributions from the US, from Australia and from some other countries outside Europe. Especially in the field of forensic child and adolescent psychiatry, which is very much influenced by legal regulations in different countries, we think that an interdisciplinary international exchange is very important to improve care and rehabilitation of these youth. A forensic manuscript from Theo A. H. Doreleijers group was the first manuscript ever published in *CAPMH *[8]. As many other adolescent forensic manuscripts published since it attracted thousand of downloads proofing thereby that online publishing is especially appropriate when dealing with interdisciplinary subjects like forensic issues. Normally layers, sociologists and others can't afford to subscribe medical journals. But if they have easy access via Pub Med these manuscripts get a much broader reception and better citations compared to publications in the traditional child psychiatry journals. Therefore we would like to invite all authors from the forensic field in child and adolescent mental health dealing with aspects of victimology or dealing with the perpetrators to submit their research in *CAPMH*.

Jörg M. Fegert and Theo A. H. Doreleijers
